# Supplementary material for: A Global Screen for Assembly State Changes of the Mitotic Proteome by SEC-SWATH-MS
Source: Cell Syst. 2020 Feb 26;10(2):133–155.e6. doi: 10.1016/j.cels.2020.01.001 (PMC7042714; doi:10.1016/j.cels.2020.01.001)

Q8WWM7 | ATX2L_HUMAN | ATXN2L A2D A2LG A2LP A2RP
Monomer MW [kDa]: 113.374 Monomer expected elution fraction: 39

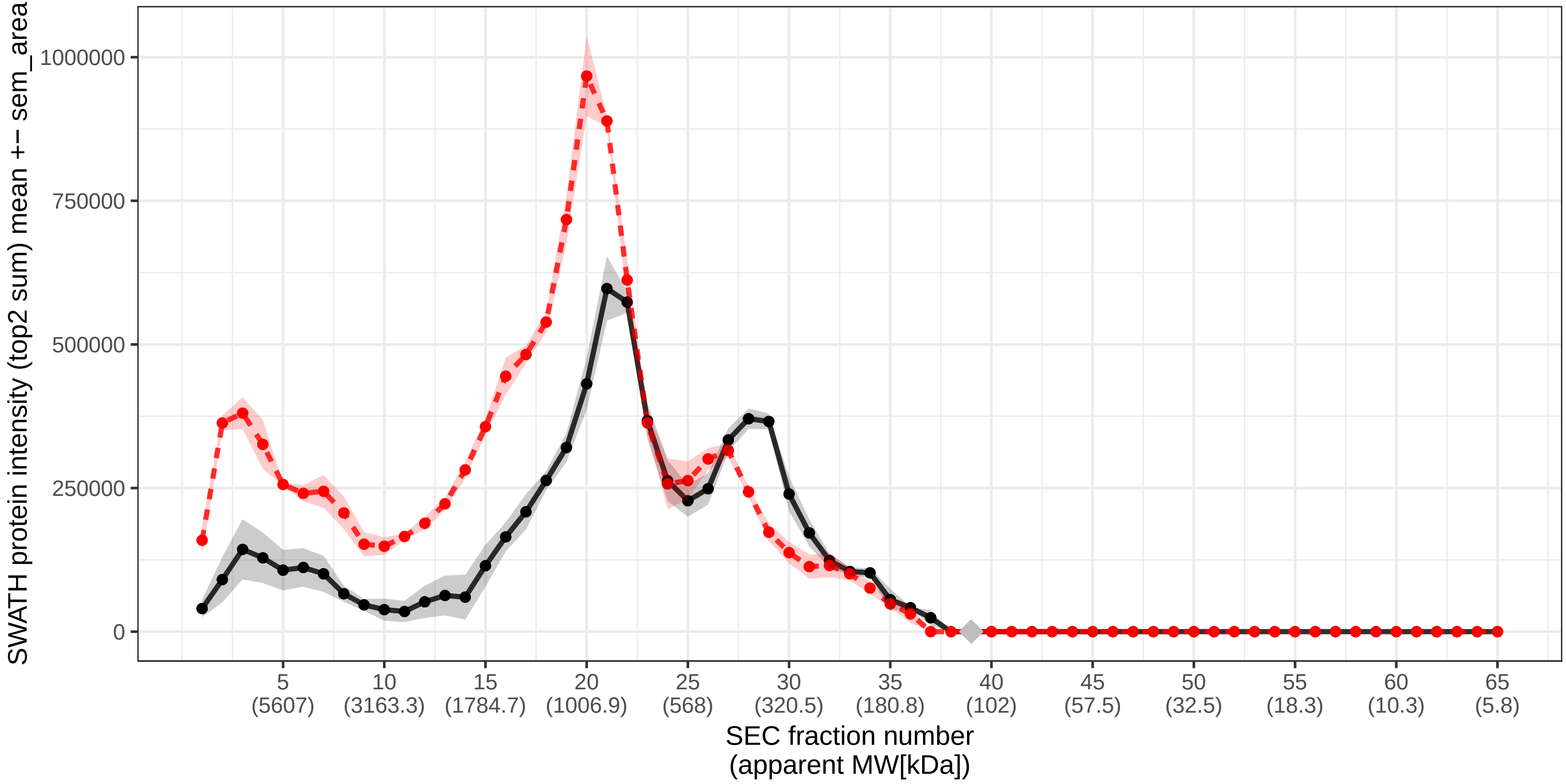

Supplement: Data S1. SEC-SWATH-MS Protein Chromatograms, Related to Figure 1 [file mmc6.zip › SECchrom_Q8WWM7_ATX2L_HUMAN_ATXN2L_A2D_A2LG_A2LP_A2RP.pdf]
